# Supplementary figures and images for: Atomic structures and deletion mutant reveal different capsid-binding patterns and functional significance of tegument protein pp150 in murine and human cytomegaloviruses with implications for therapeutic development
Source: PLoS Pathog. 2019 Feb 19;15(2):e1007615. doi: 10.1371/journal.ppat.1007615 (PMC6396938; doi:10.1371/journal.ppat.1007615)

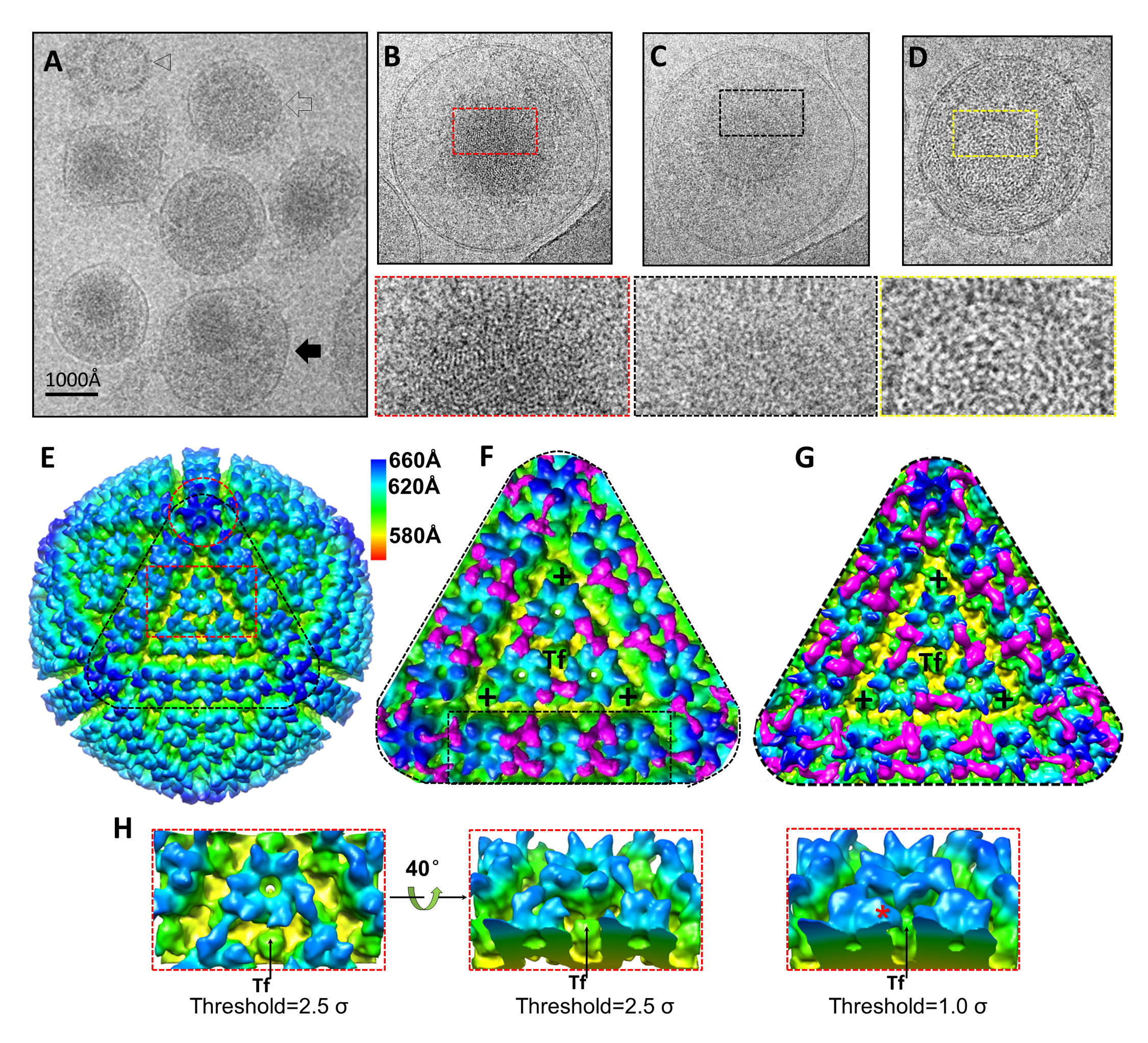

Supplement: S1 Fig — (A) A cryoEM micrograph of wild-type MCMV, containing virions (solid black arrow), a non-infectious enveloped particle (NIEP, open black arrow), and a naked capsid (black arrowhead). (B-D) Representative enveloped MCMV particles with DNA (B; i.e., virion), without DNA (C; i.e., NIEP), and with leaked out DNA (D). A characteristic “finger-print” pattern of closely packed viral DNA is visible in (B), not in the NIEP (C). Presumably, a portion of DNA can leak out from a structurally-compromised capsid, a characteristic fingerprint pattern of closely packed DNA becomes loose and “spaghetti-like”, spreading out into space beyond the capsid within the virion (D). These DNA features are shown more clearly in the zoom-in views of the upper portion of the capsid at the bottom of each panel. (E) Radially colored surface representation of the 3D icosahedral reconstruction of the wild-type MCMV virion at ~12 Å resolution, viewed along a 3-fold axis. (F-G) Enlargement of a facet (F) [the triangle region in (E)] and that of the corresponding area in the reconstruction of the detergent-treated virions (i.e., Fig 1B low-passed filtered to 12 Å resolution) (G) showing the “Λ”-shaped tegument proteins (magenta) connecting triplexes, hexons, and pentons. (H) The boxed area of (E) observed from two different views and at two different density thresholds. Tegument densities (one marked by an asterisk) are visible and are associated with triplex Tf in the right panel when the map is rendered at a threshold of 1.0 standard deviation (σ). (TIF) [file ppat.1007615.s001.tif]

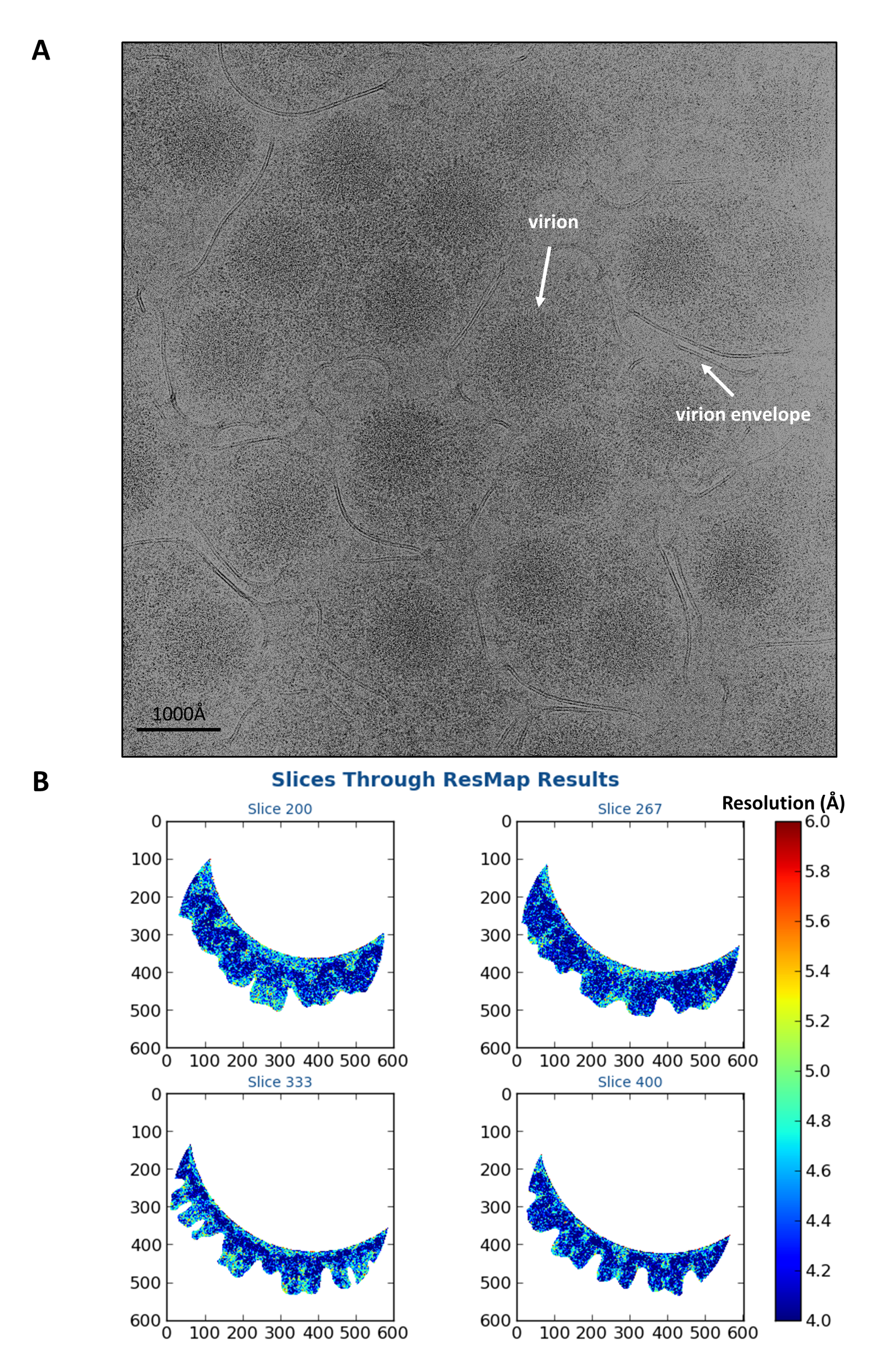

Supplement: S2 Fig — (A) Image recorded on photographic film shows a high viral particle concentration. (B) Local resolution heat maps of representative density slices through an asymmetric unit, obtained with ResMap [28]. Color scheme for local resolutions is shown in the color bar. (TIF) [file ppat.1007615.s002.tif]

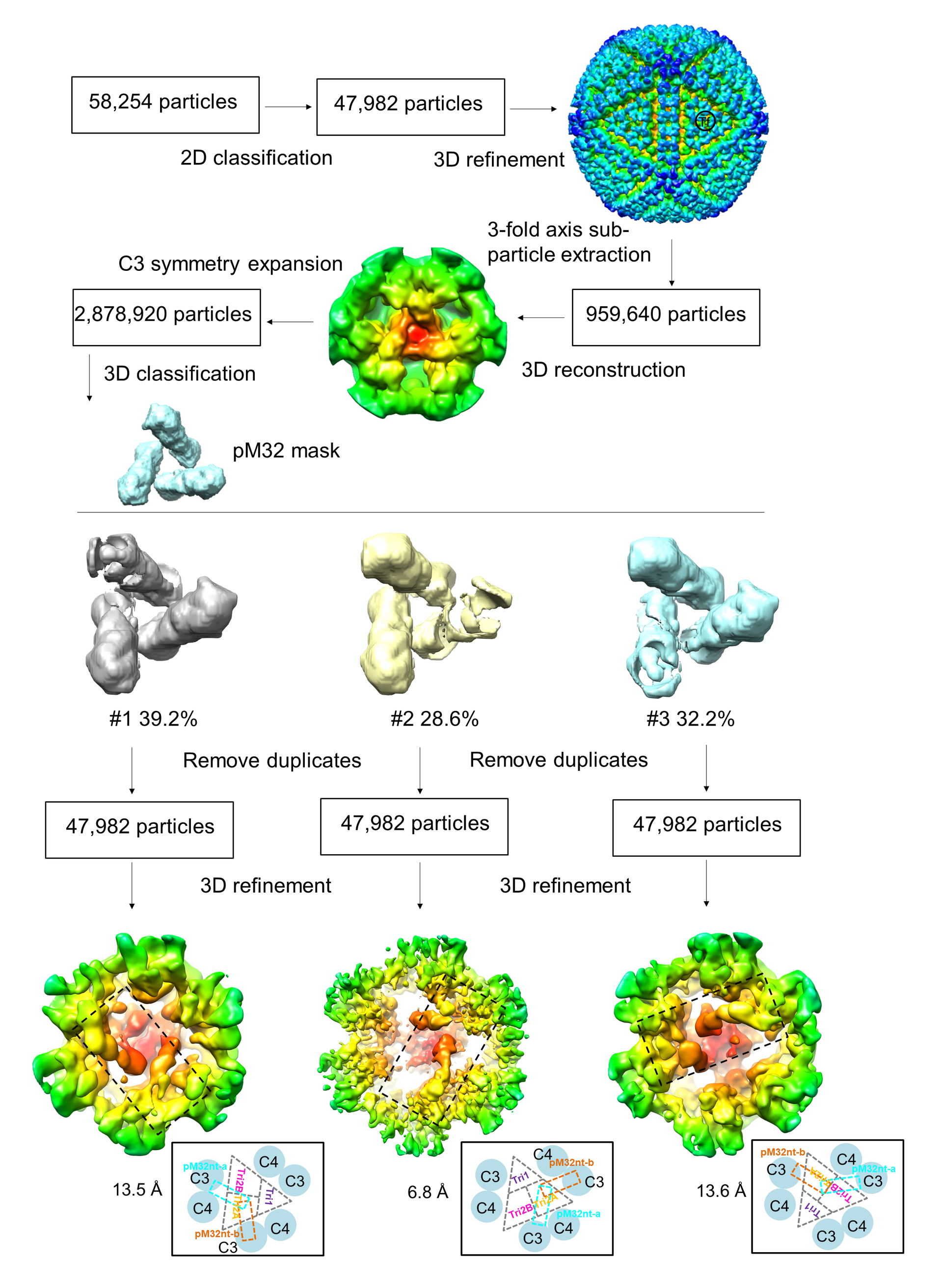

Supplement: S3 Fig — Viral particles were sorted out and subjected to 2D classification in Relion [55] to select only virion particles for icosahedral reconstruction. Sub-particles containing tegument contributions above triplex Tf were extracted with a 3-fold symmetric pp150 mask and subjected to 3D classification and refinement. All the resulting reconstructions contain only two pM32 subunits. (TIF) [file ppat.1007615.s003.tif]

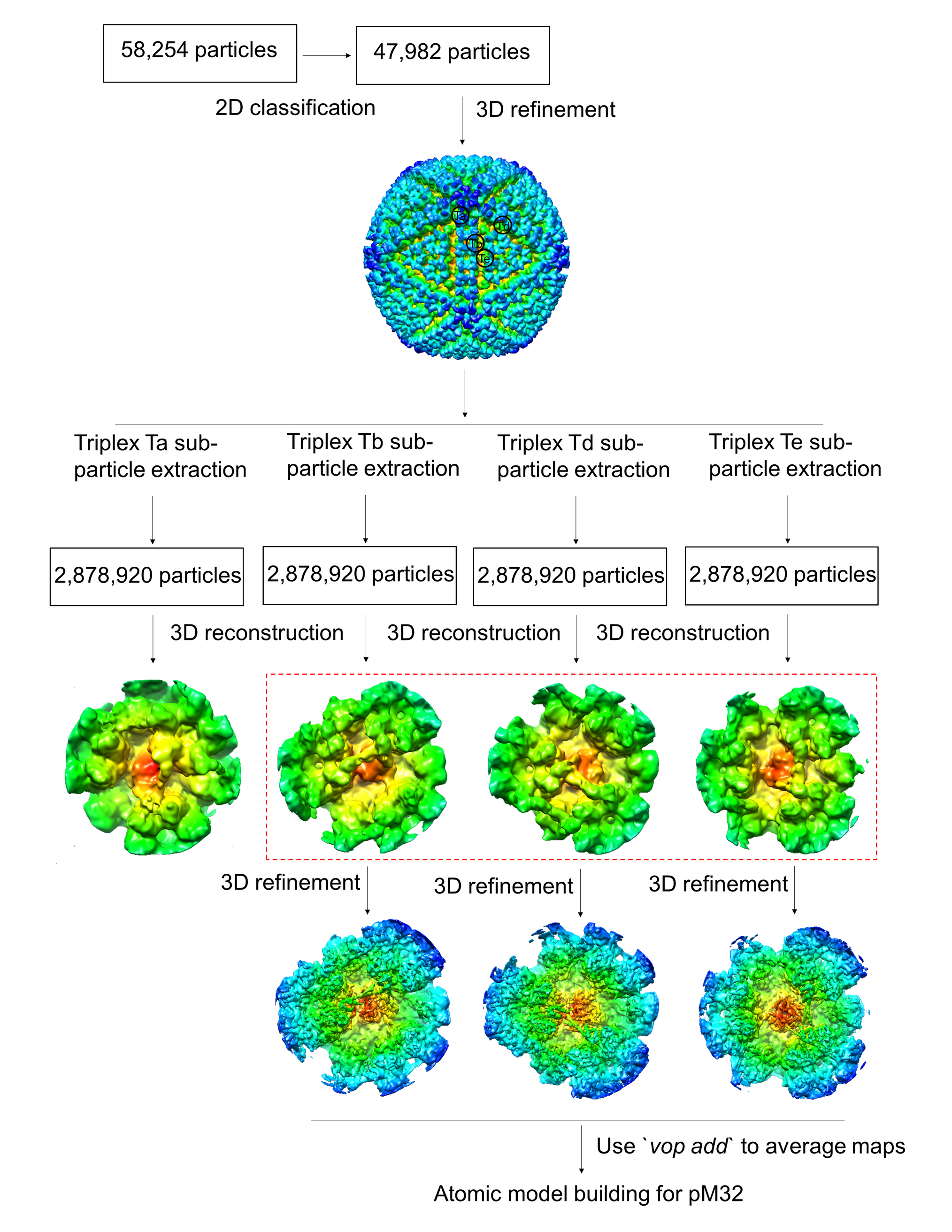

Supplement: S4 Fig — Sub-particles from triplex regions (Ta, Tb, Td, and Te) were extracted and reconstructed separately. Sub-particles from triplex Tb, Td, and Te regions were further refined and averaged by ‘vop’ tool in Chimera to boost the signal-to-noise ratio of the density map. pM32 densities in the resulting averaged map was used for atomic model building. (TIF) [file ppat.1007615.s004.tif]

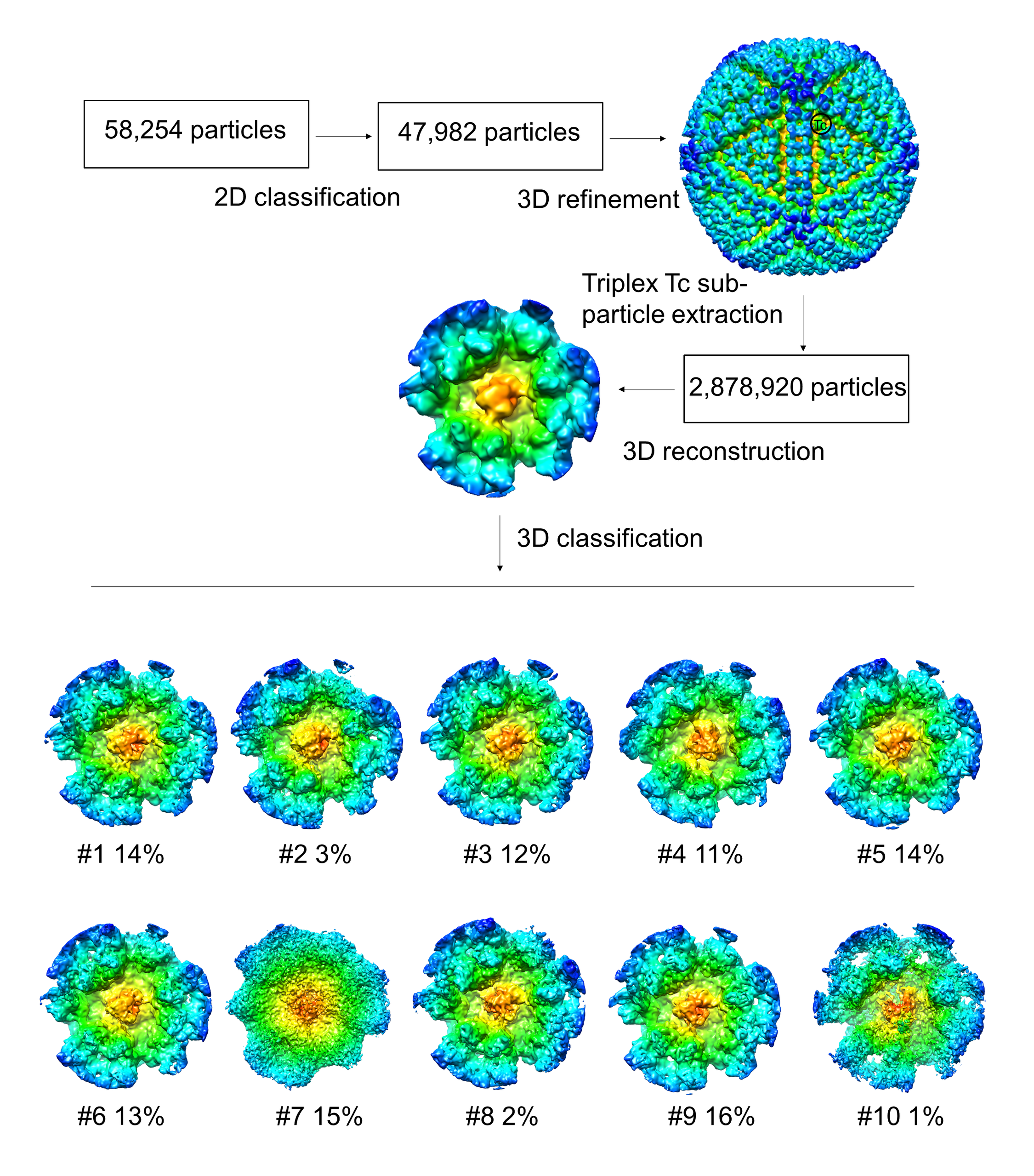

Supplement: S5 Fig — Sub-particles from triplex Tc region were extracted and processed by 3D classification to confirm the absence of pM32 on triplex Tc. (TIF) [file ppat.1007615.s005.tif]

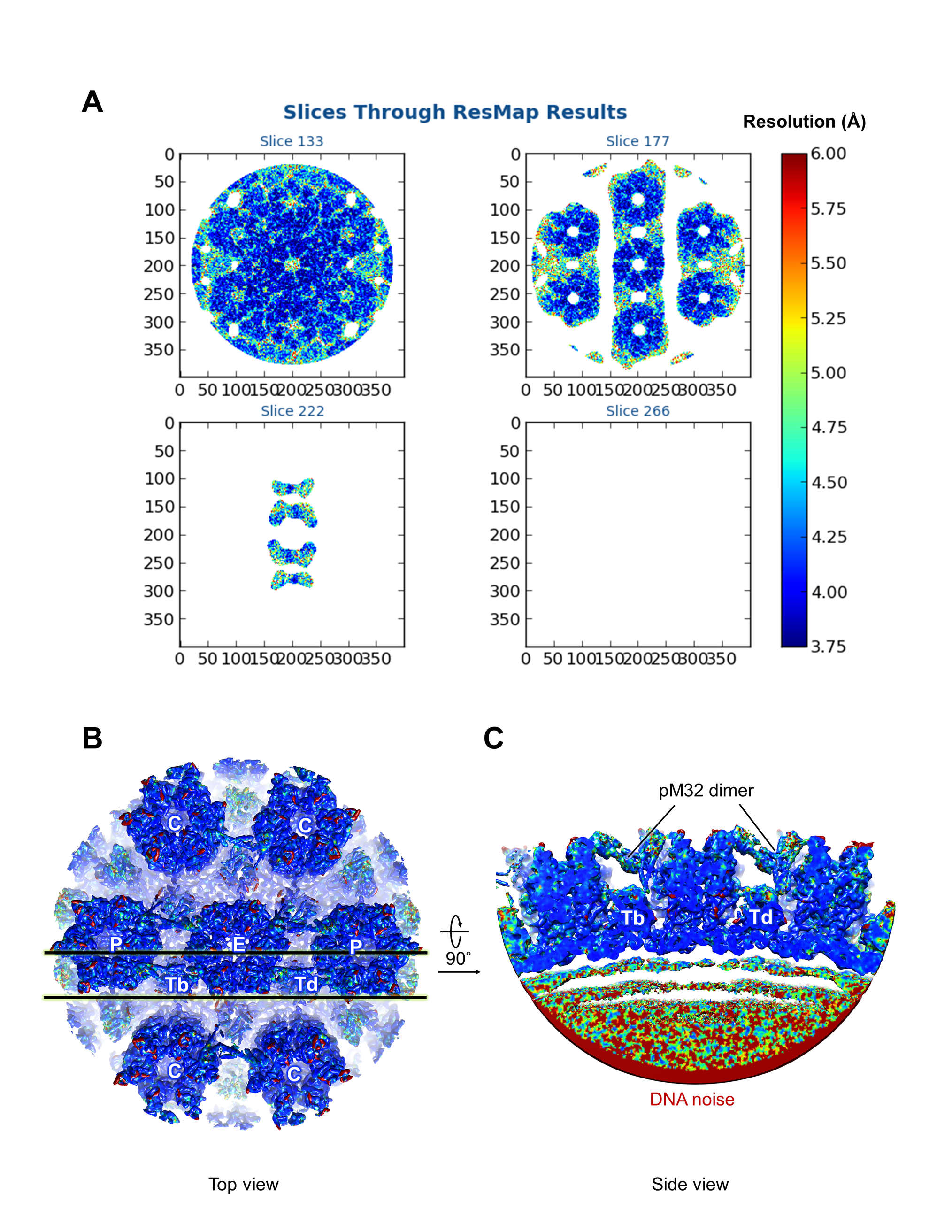

Supplement: S6 Fig — Representative slices (A) and surface views (B, C) of the sub-particle reconstruction showing local resolution heat maps generated by ResMap [28]. The side view in (C) only shows the density slab demarked by the two horizontal lines in the top view (B). Color scheme for local resolutions is shown in the color bar. Hexons C, E, P and triplexes Tb, Td are labeled. (TIF) [file ppat.1007615.s006.tif]

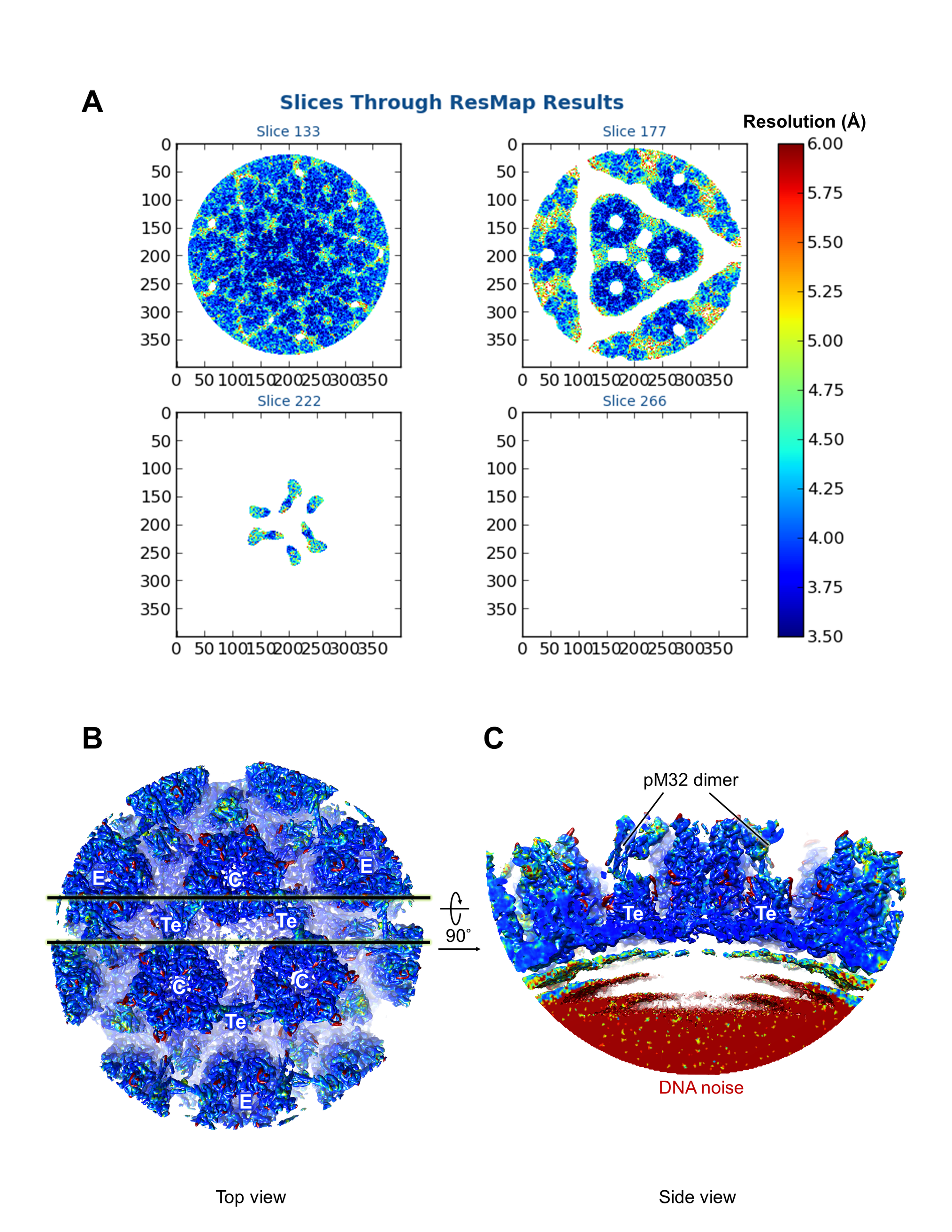

Supplement: S7 Fig — Representative slices (A) and surface views (B, C) of the sub-particle reconstruction showing local resolution heat maps generated by ResMap [28]. The side view in (C) only shows the density slab demarked by the two horizontal lines in the top view (B). Color scheme for local resolutions is shown in the color bar. Hexons C, E and triplexes Te are labeled. (TIF) [file ppat.1007615.s007.tif]

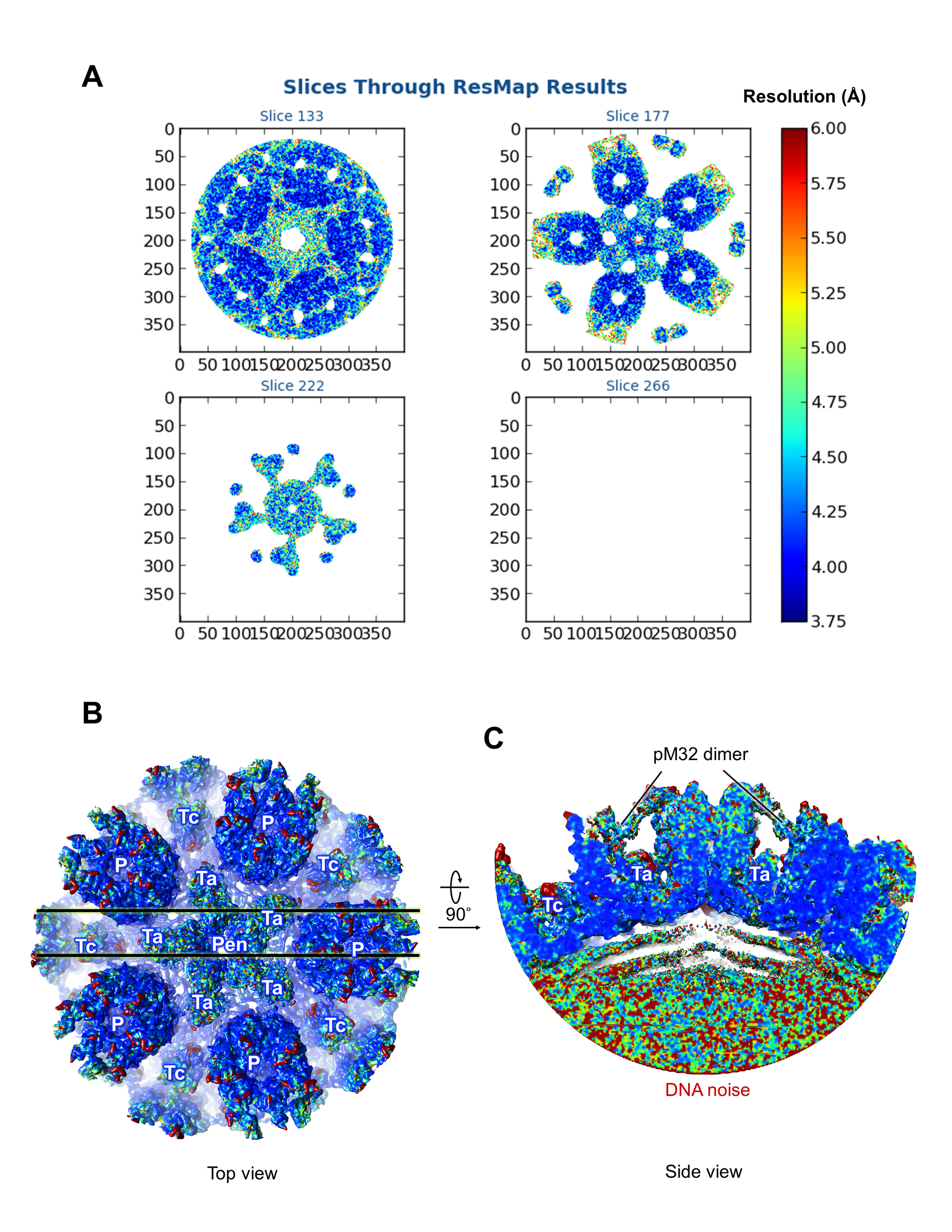

Supplement: S8 Fig — Representative slices (A) and surface views (B, C) of the sub-particle reconstruction showing local resolution heat maps generated by ResMap [28]. The side view in (C) only shows the density slab demarked by the two horizontal lines in the top view (B). Color scheme for local resolutions is shown in the color bar. Penton, Hexons P, and triplexes Ta, Tc are labeled. (TIF) [file ppat.1007615.s008.tif]

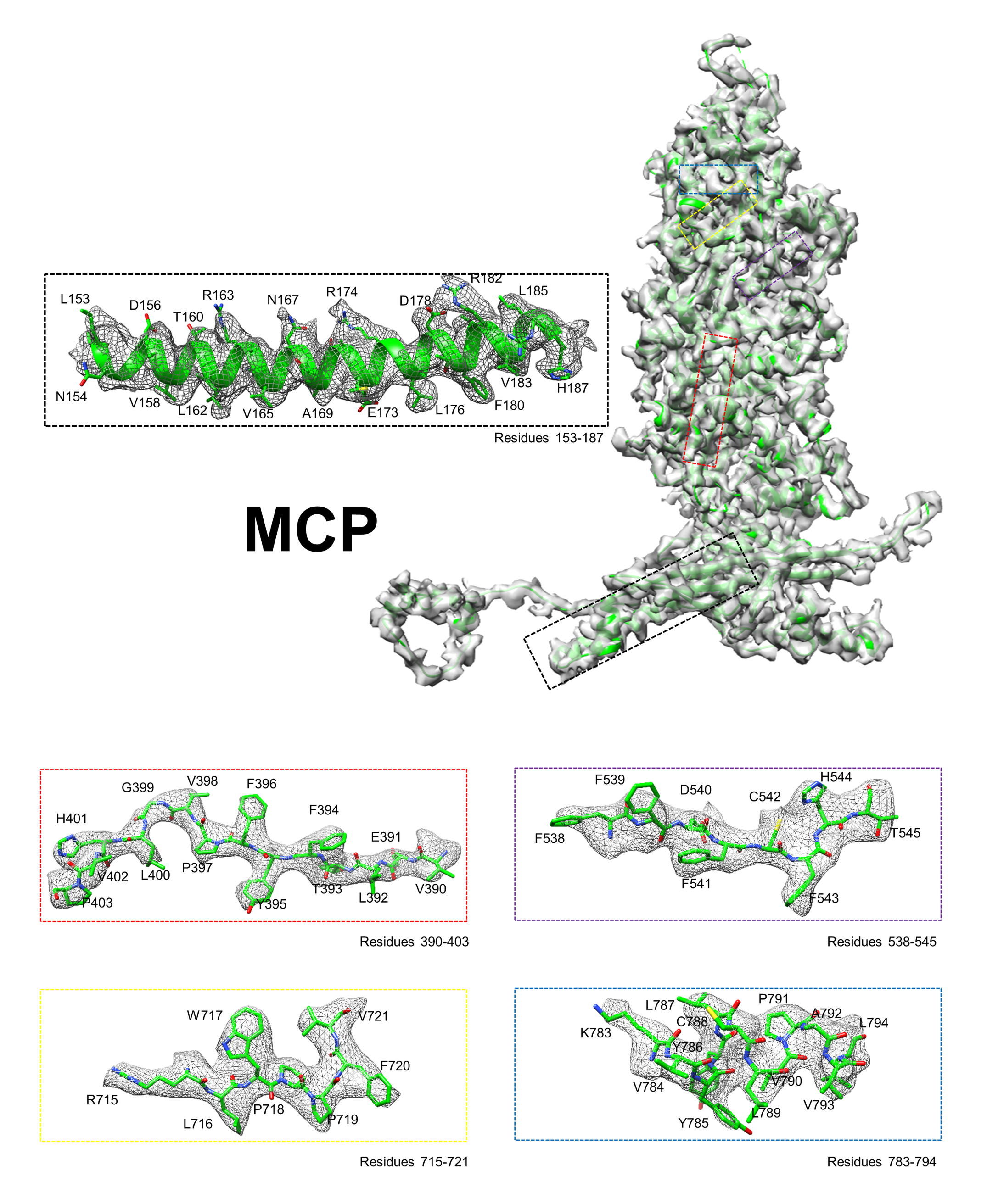

Supplement: S9 Fig — The density map (gray) of a hexon MCP segmented out from the 3-fold axis sub-particle reconstruction (3.6 Å) is superposed with its atomic model (ribbon). Boxed regions are enlarged with density shown as gray mesh and atomic models as ribbon/sticks in the boxes with corresponding color edges. (TIF) [file ppat.1007615.s009.tif]

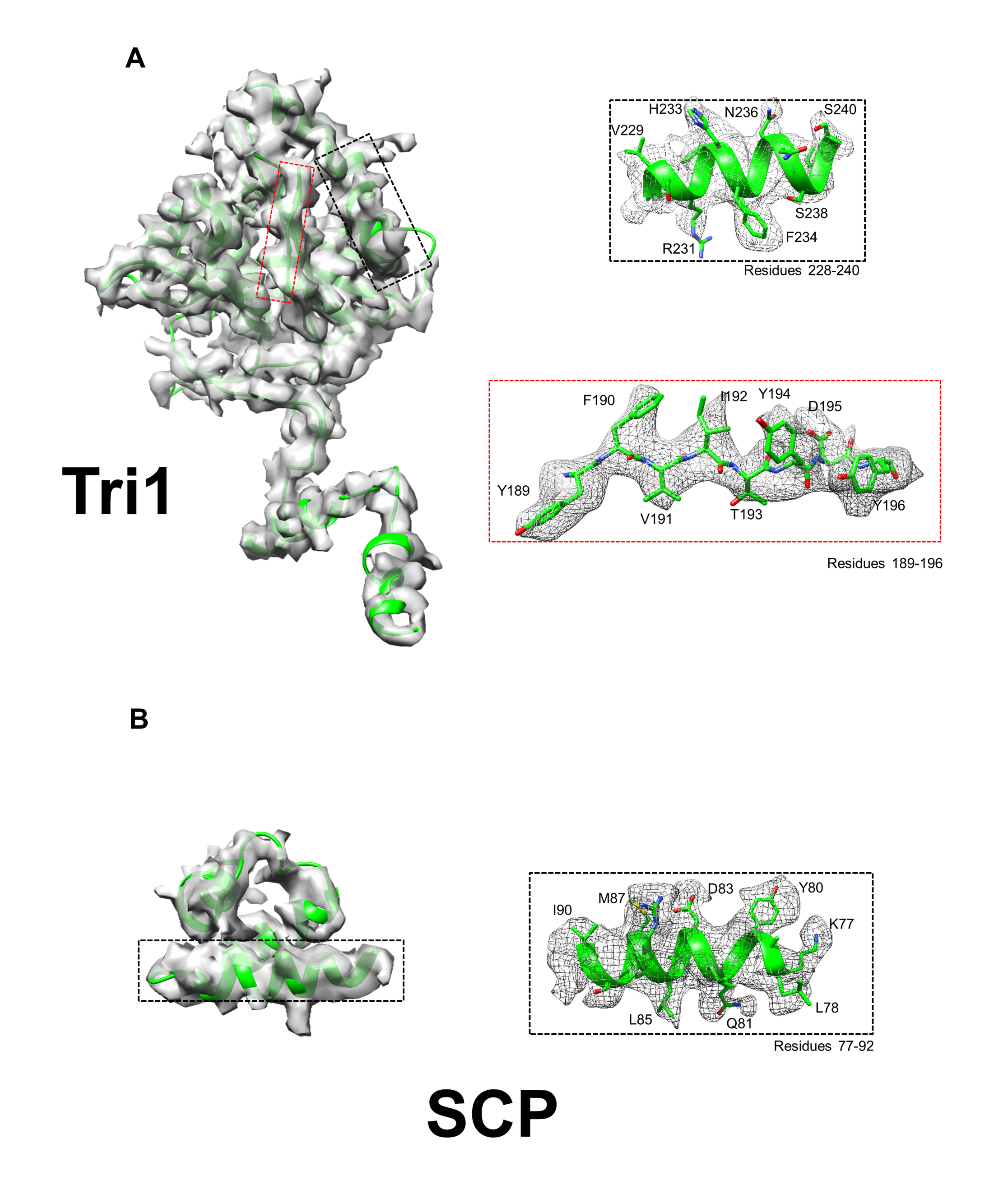

Supplement: S10 Fig — (A-B) The density maps (gray) of a Tri1 (A) and an SCP (B) segmented out from the 3-fold axis sub-particle reconstruction (3.6 Å) are superposed with their atomic models (ribbon). Boxed regions are enlarged with density shown as gray mesh and atomic models as ribbon/sticks in the boxes with corresponding color edges. (TIF) [file ppat.1007615.s010.tif]

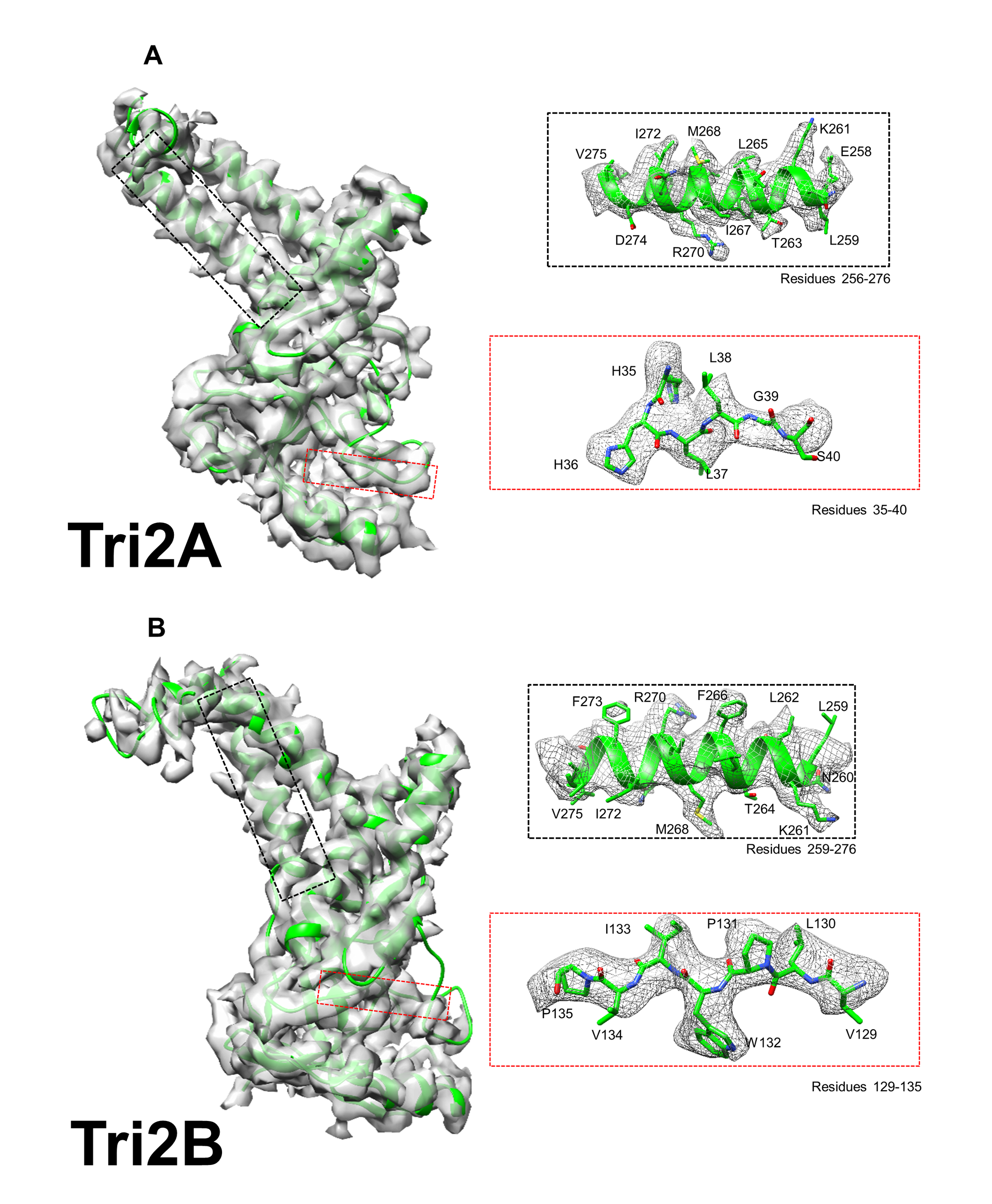

Supplement: S11 Fig — (A-B) The density maps (gray) of a Tri2A (A) and a Tri2B (B) segmented out from the 3-fold axis sub-particle reconstruction (3.6 Å) are superposed with their atomic models (ribbon). Boxed regions are enlarged with density shown as gray mesh and atomic models as ribbon/sticks in the boxes with corresponding color edges. (TIF) [file ppat.1007615.s011.tif]

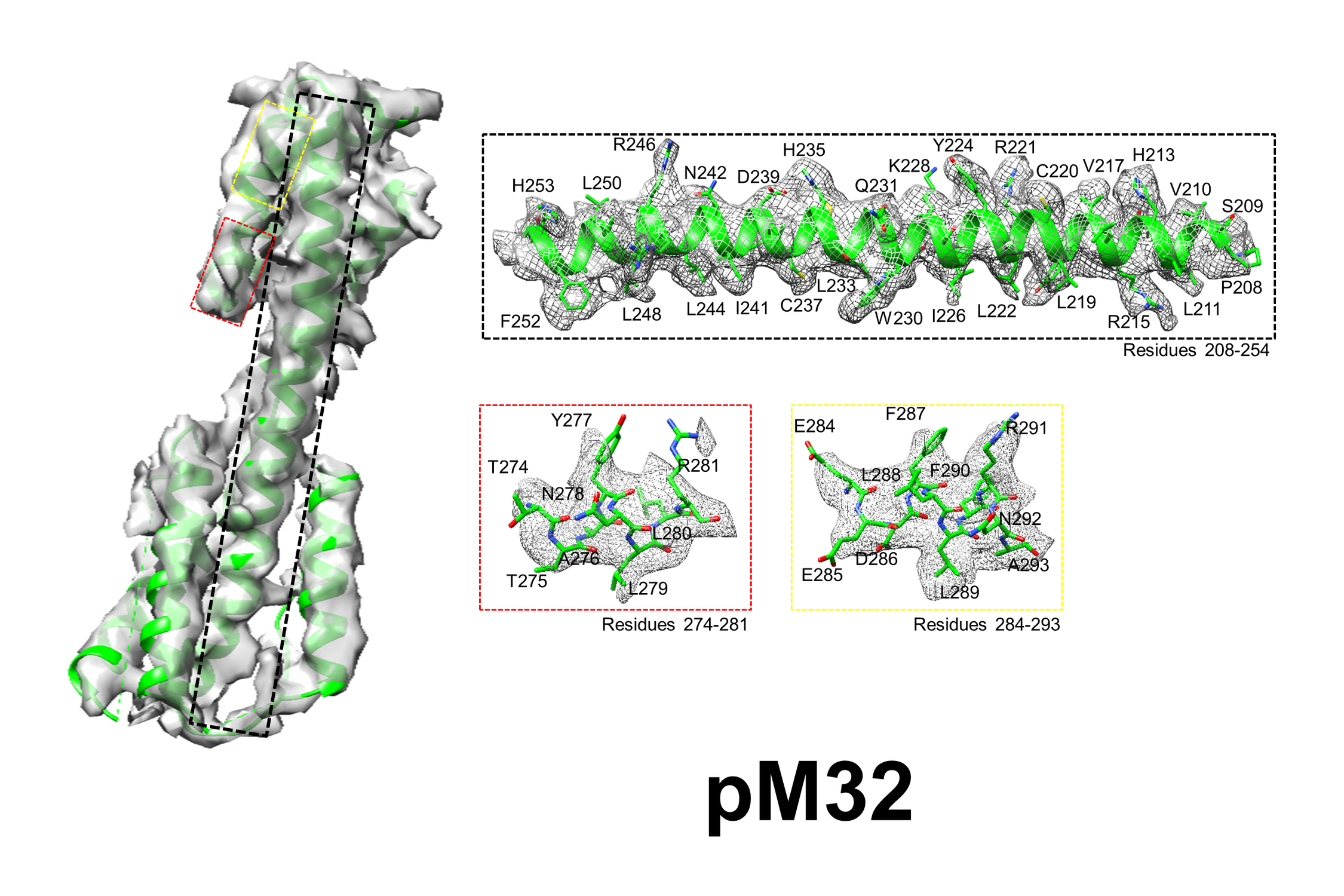

Supplement: S12 Fig — The density map (gray) of a pM32 segmented out from the 3-fold axis sub-particle reconstruction (3.6 Å) is superposed with its atomic model (ribbon). Boxed regions are enlarged with density shown as gray mesh and atomic models as ribbon/sticks in the boxes with corresponding color edges. (TIF) [file ppat.1007615.s012.tif]

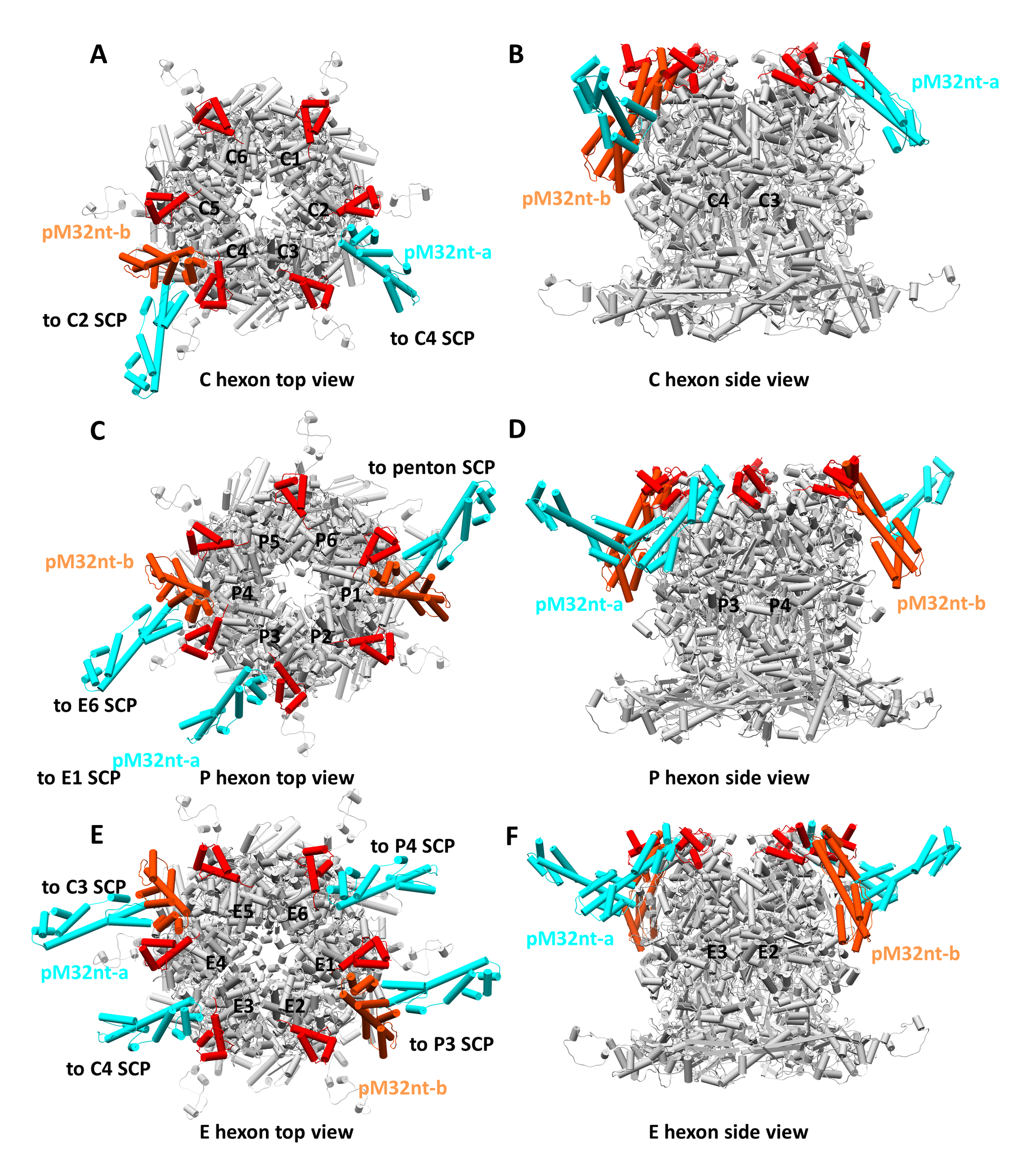

Supplement: S13 Fig — (A) Top view of a C capsomer and its interacting pM32nt subunits, showing C capsomer is stabilized by three copies of pM32nt. (B) Side view of (A). (C) Top view of a P capsomer and its interacting pM32nt subunits, showing P capsomer is stabilized by five copies of pM32nt. (D) Side view of (C). (E) Top view of an E capsomer and its interacting pM32nt subunits, showing E capsomer is stabilized by six copies of pM32nt. (F) Side view of (E). (TIF) [file ppat.1007615.s013.tif]

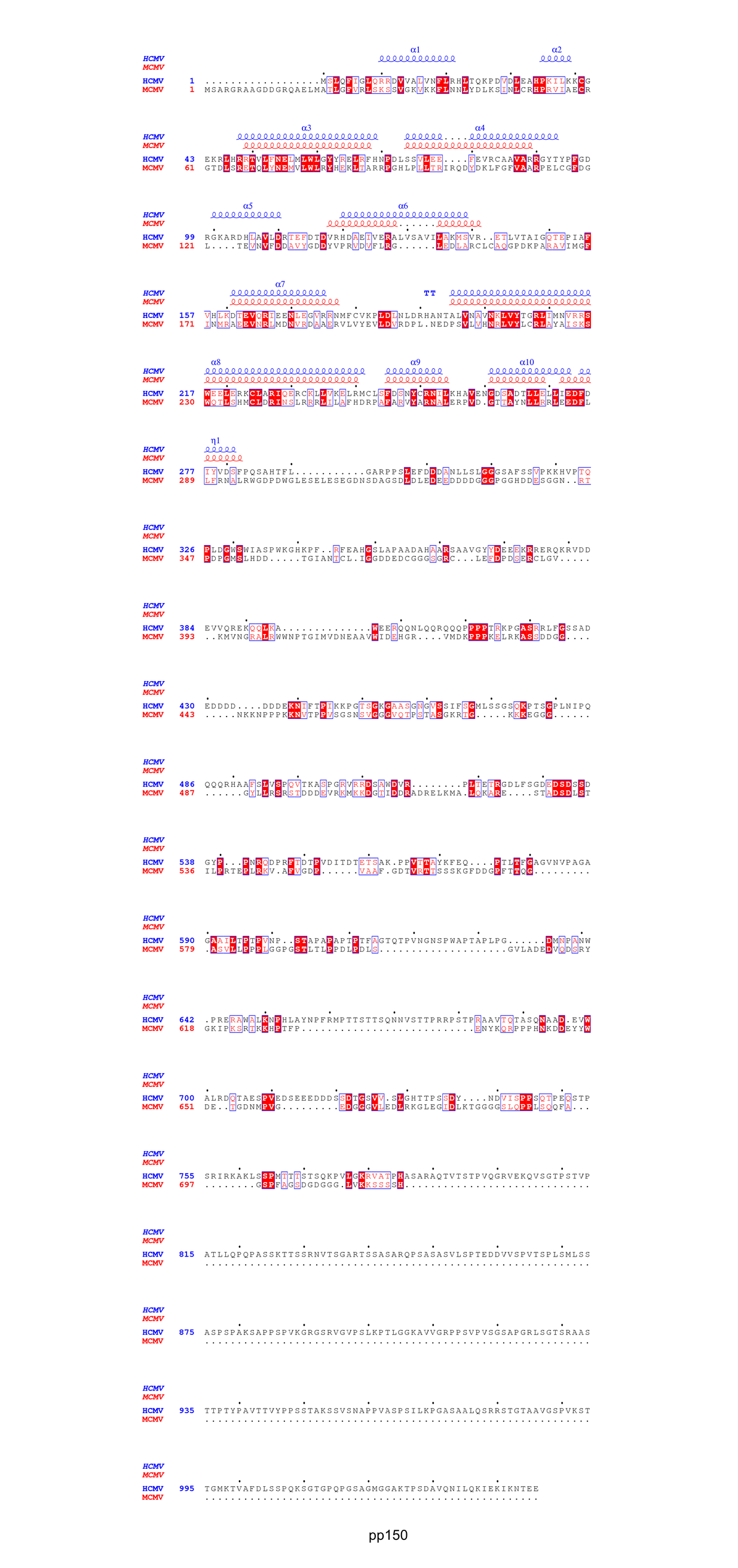

Supplement: S14 Fig — Schematic representations of the amino acid sequence and secondary structure alignment for pUL32nt and pM32nt in HCMV and MCMV analyzed and displayed by ESPript 3.0 [66]. Spiral represents α-helix. (TIF) [file ppat.1007615.s014.tif]

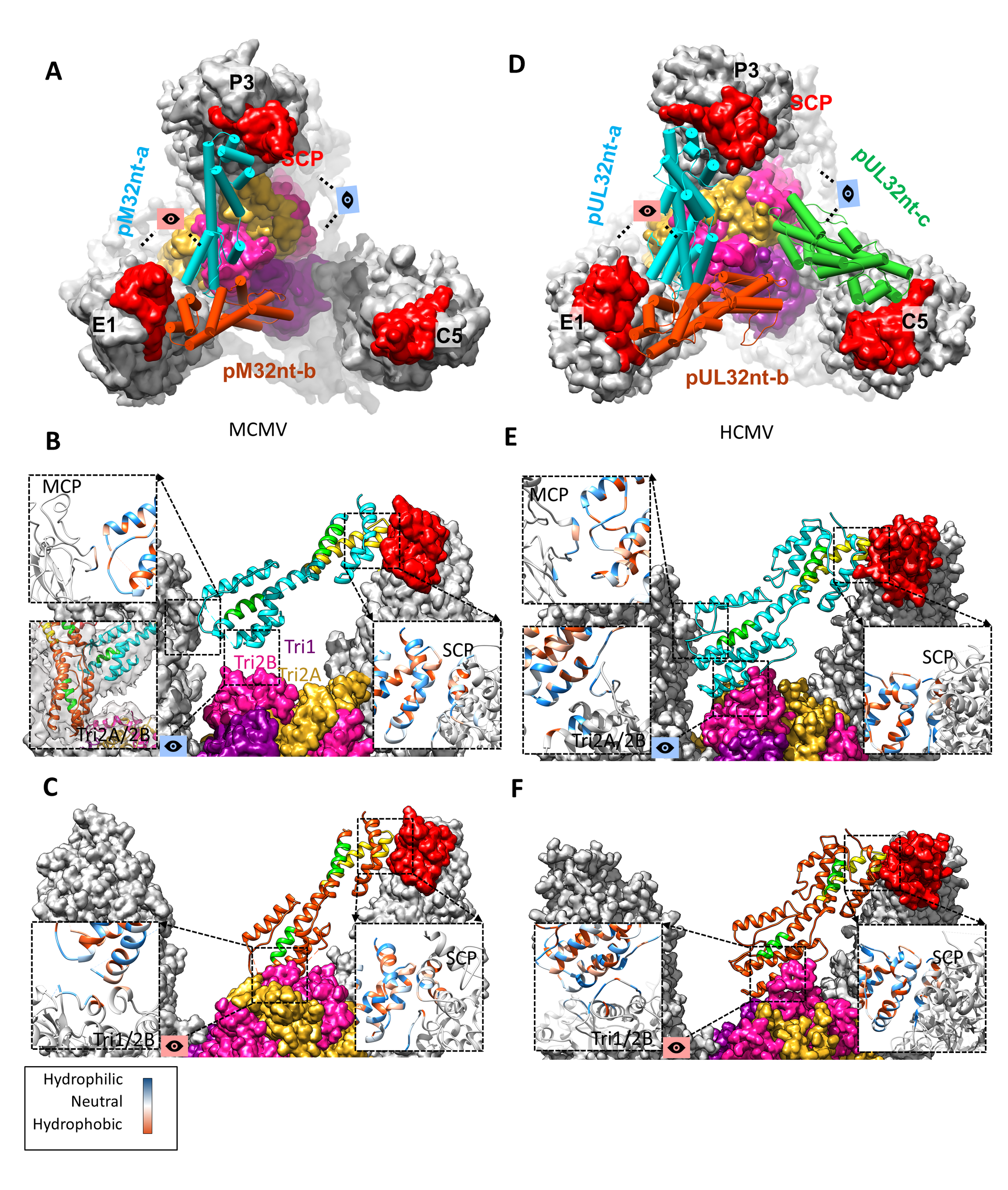

Supplement: S15 Fig — (A) Top view (down a local 3-fold axis) of triplex Tb region showing a pM32 dimer—pM32nt-a (cyan) and pM32nt-b (orange red)—bound to the triplex and extending towards the SCPs atop nearby MCPs in MCMV. (B-C) Side views of the structure in (A), showing how pM32nt-a (B) and pM32nt-b (C) interact with capsid proteins. (D-F) Corresponding views in HCMV. (TIF) [file ppat.1007615.s015.tif]

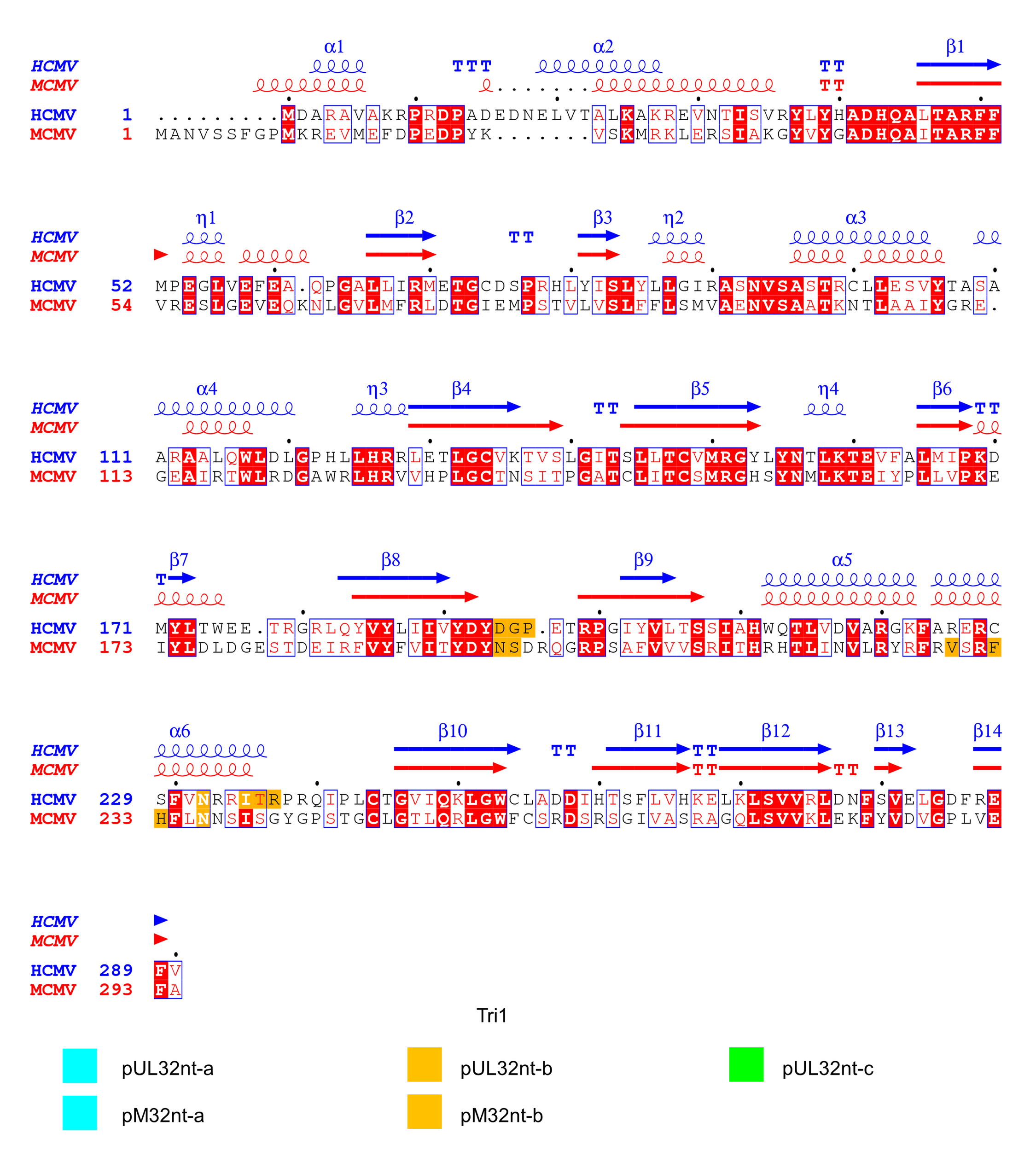

Supplement: S16 Fig — Schematic representations of the amino acid sequence and secondary structure alignment for Tri1 in HCMV and MCMV analyzed and displayed by ESPript 3.0 [66]. Arrow and spiral represent β-sheet and α-helix, respectively. Residues in Tri1 interacting with pUL32-a/pM32-a (cyan), pUL32-b/pM32-b (orange red), and pUL32-c (green, only in HCMV) are colored. (TIF) [file ppat.1007615.s016.tif]

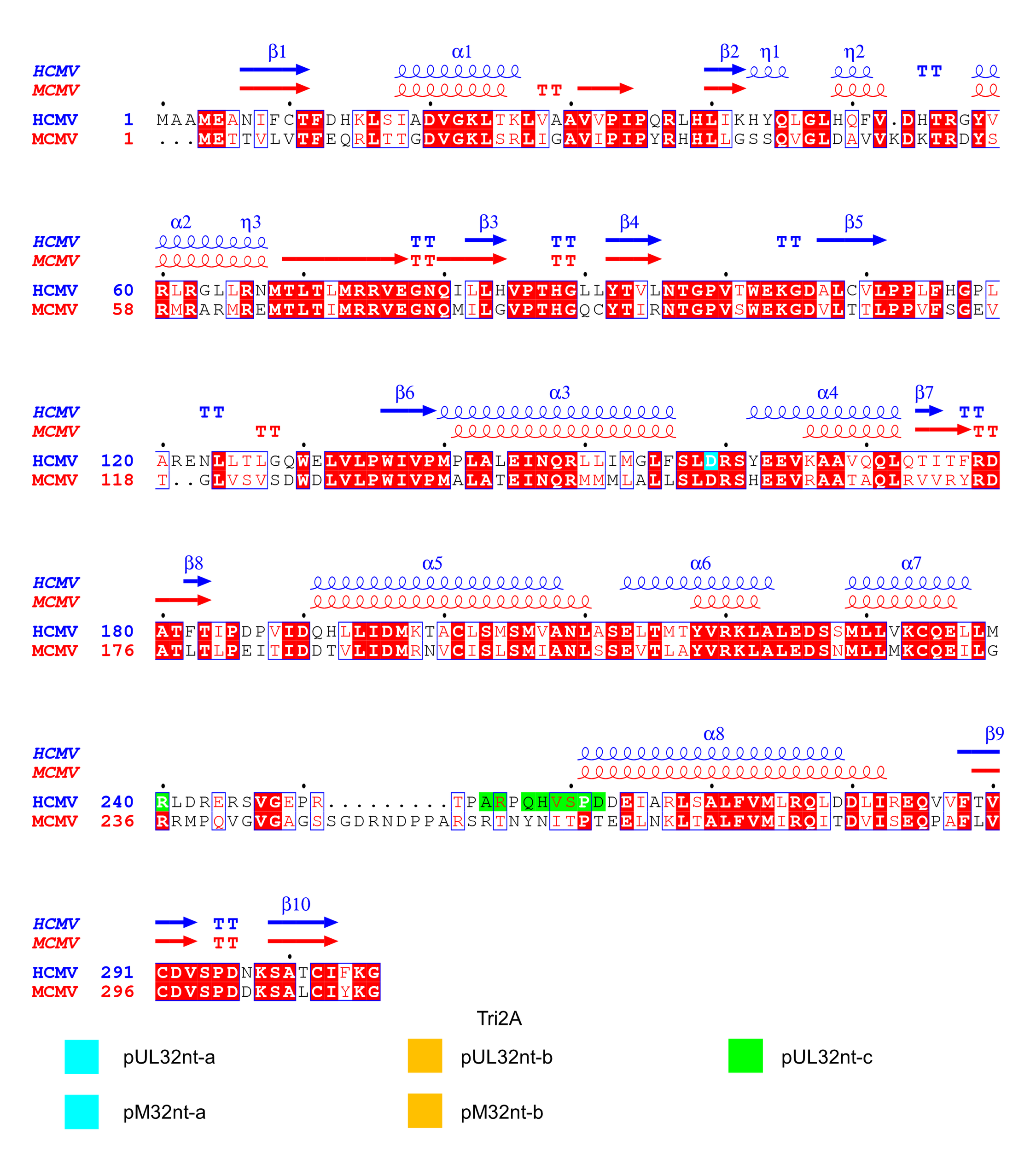

Supplement: S17 Fig — Schematic representations of the amino acid sequence and secondary structure alignment for Tri2A in HCMV and MCMV analyzed and displayed by ESPript 3.0 [66]. Arrow and spiral represent β-sheet and α-helix, respectively. Residues in Tri2A interacting with pUL32-a/pM32-a (cyan), pUL32-b/pM32-b (orange red), and pUL32-c (green, only in HCMV) are colored. (TIF) [file ppat.1007615.s017.tif]

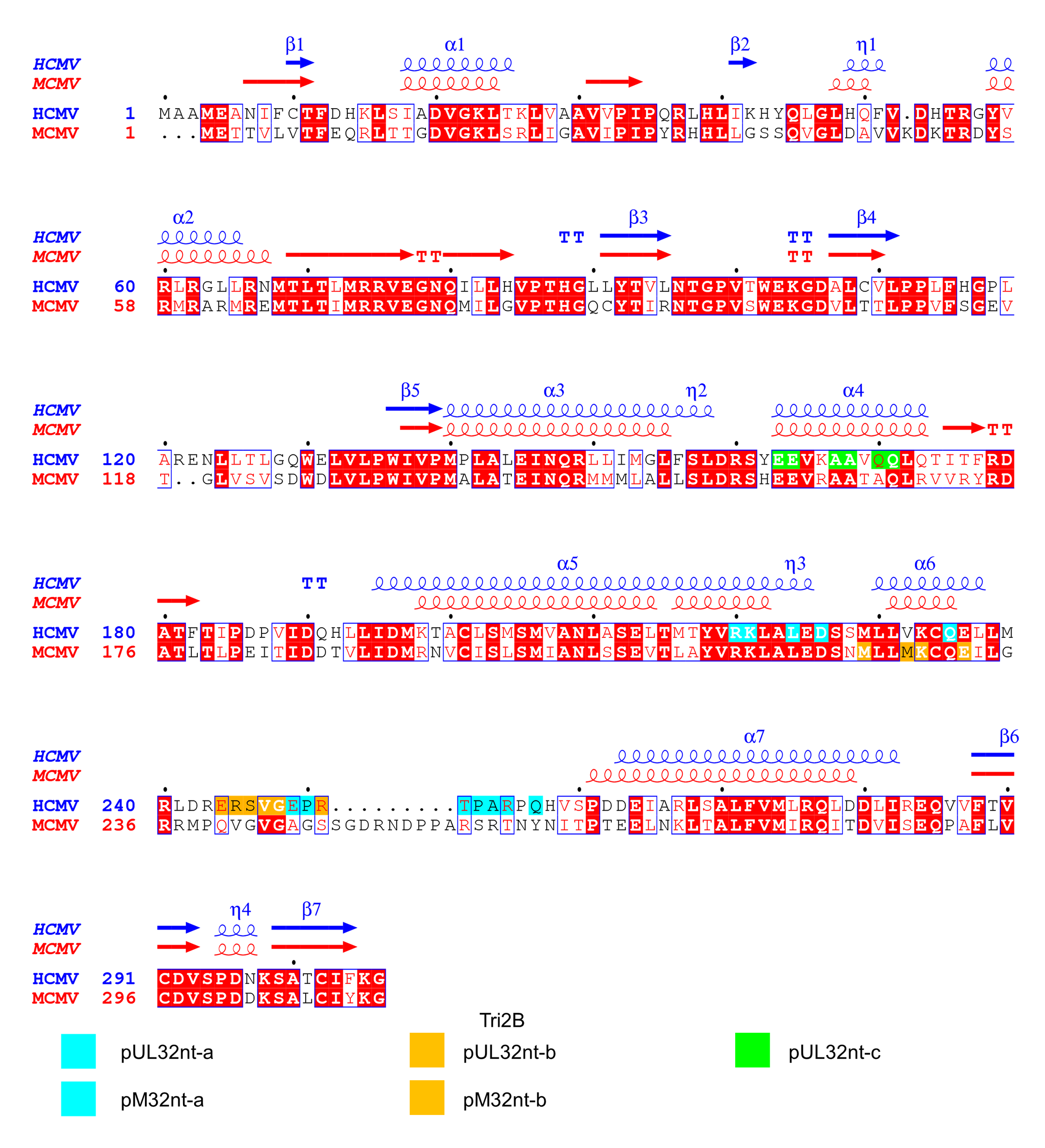

Supplement: S18 Fig — Schematic representations of the amino acid sequence and secondary structure alignment for Tri2B in HCMV and MCMV analyzed and displayed by ESPript 3.0 [66]. Arrow and spiral represent β-sheet and α-helix, respectively. Residues in Tri2B interacting with pUL32-a/pM32-a (cyan), pUL32-b/pM32-b (orange red), and pUL32-c (green, only in HCMV) are colored. (TIF) [file ppat.1007615.s018.tif]

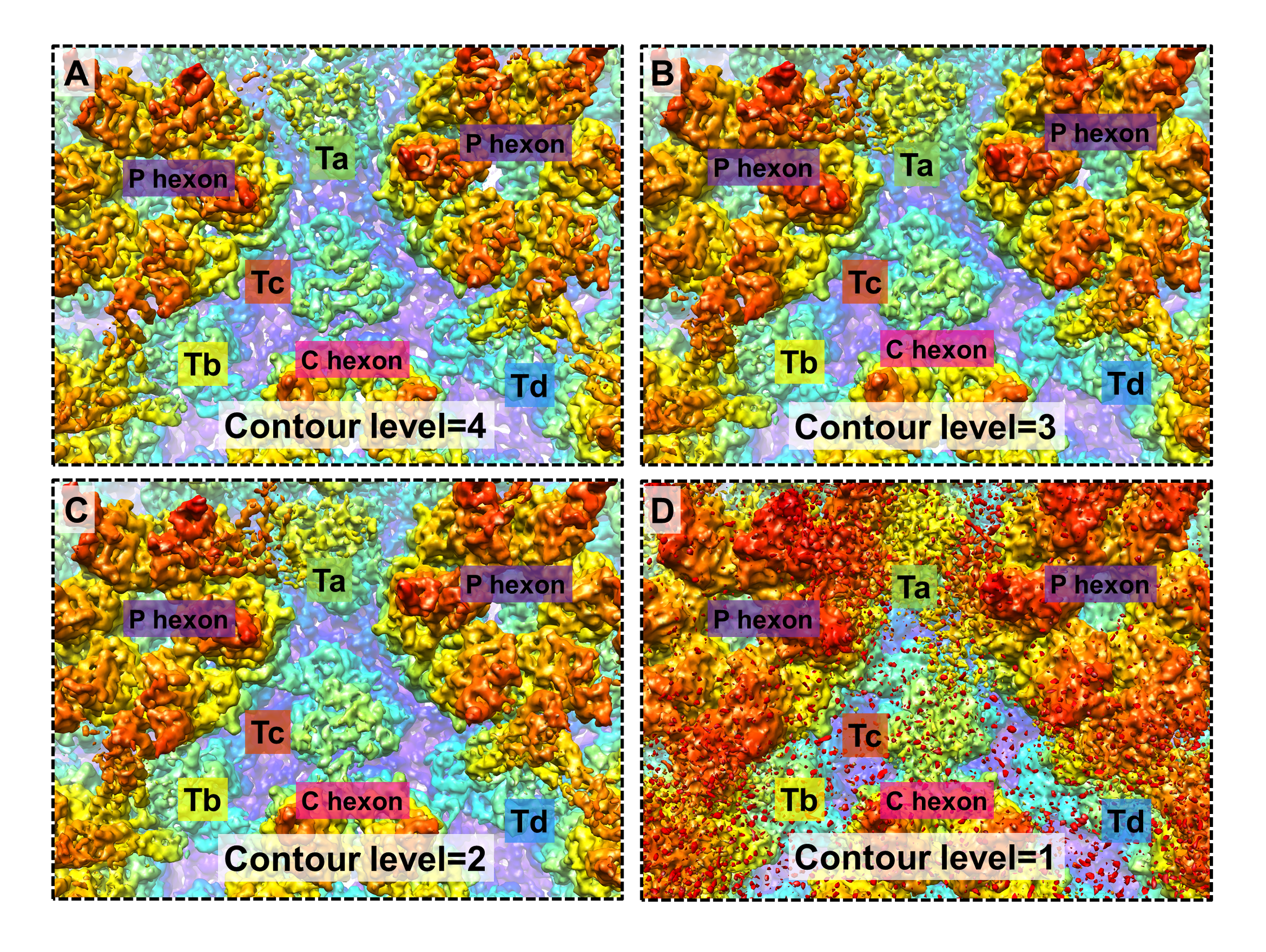

Supplement: S19 Fig — (TIF) [file ppat.1007615.s019.tif]

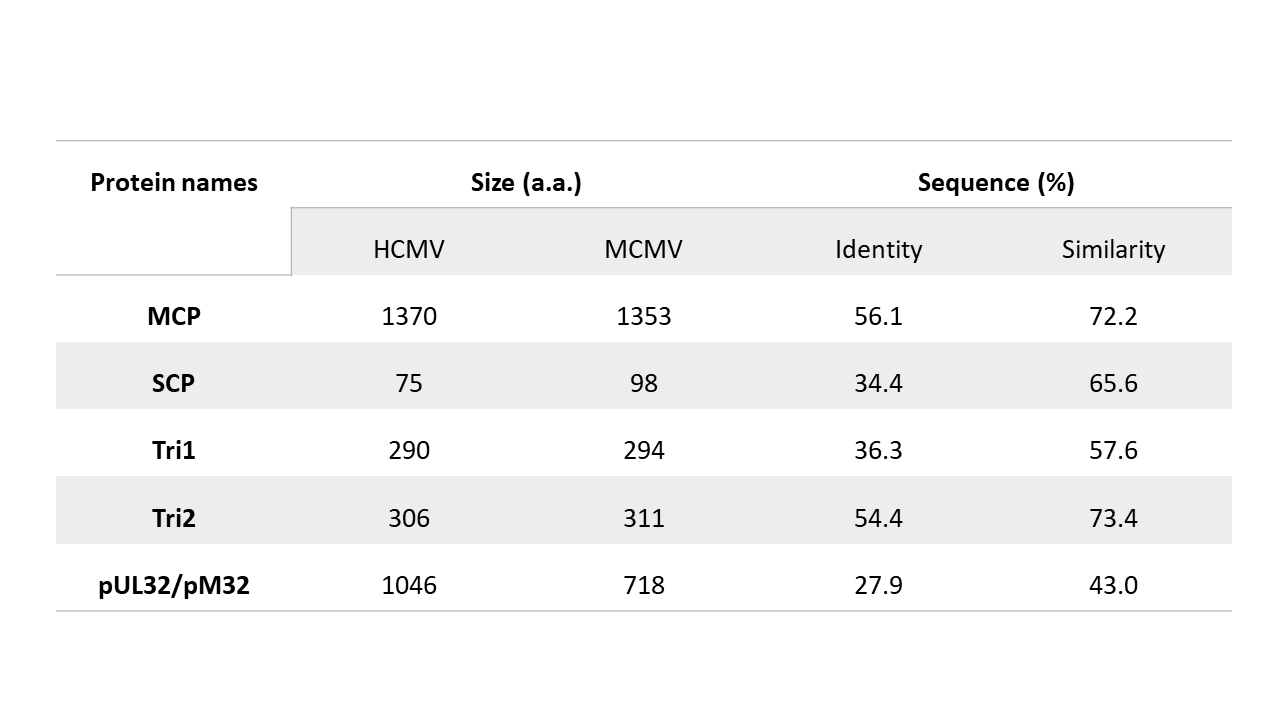

Supplement: S1 Table — (TIF) [file ppat.1007615.s020.tif]

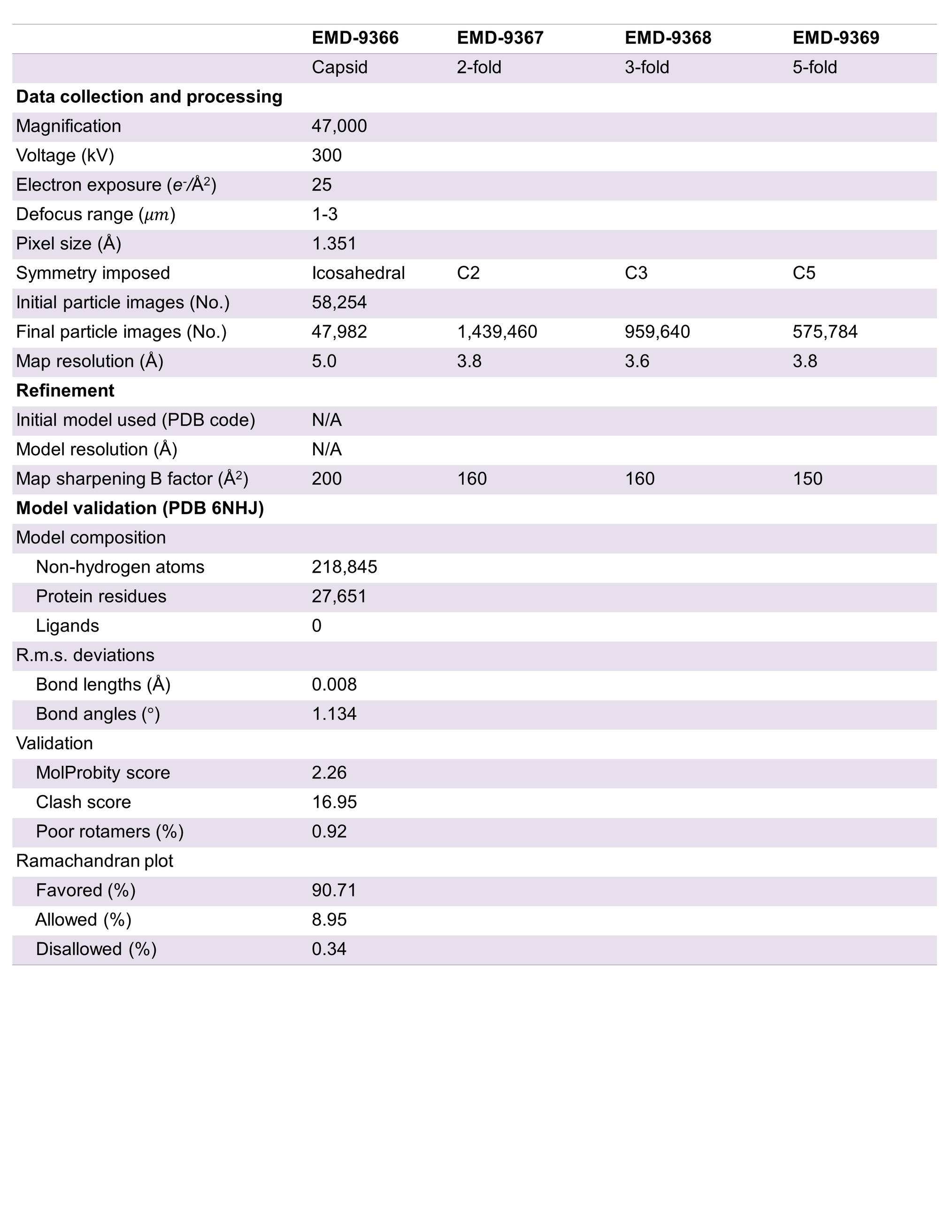

Supplement: S2 Table — (TIF) [file ppat.1007615.s021.tif]
